# The challenge of one billion adjuvanted vaccine doses: evaluating scalability, sustainability, and supply capacity of Quillaja saponin QS-21 for large-scale vaccine demand

**DOI:** 10.3389/fimmu.2026.1813847

**Published:** 2026-04-17

**Authors:** Leandro Padilla, Roberto Bobadilla-Fazzini, Marcelo Orellana, Javier González, Damian Hiley, Rodrigo Otero, Zoltan Beck

**Affiliations:** 1 Desert King Chile, Valparaíso, Chile; 2 Desert King International, San Diego, CA, United States

**Keywords:** adjuvant, QS-21, saponin, sustainable supply, vaccine

## Abstract

The biomass of *Quillaja saponaria* is a well-characterized source of triterpenic saponins, a family of amphiphilic glycosides with broad industrial and biomedical relevance. Among these, the highly purified fraction QS-21, and the related fractions QHA and QHC, have strong immunostimulant properties and have become key adjuvant components of several FDA-approved human vaccines. The expanding global use of QS-21-based adjuvants has raised concerns regarding long-term availability and sustainability, as commercial production currently relies exclusively on extraction from *Q. saponaria* biomass sourced in Chile. This review summarizes the historical development, chemical features, and immunological relevance of QS-21, and critically evaluates existing and emerging production technologies in terms of a projected production benchmark of 50 Kg per year (corresponding to at least one billion 50 µg doses). We conclude that appropriate management of wild Quillaja forests can sustain kilogram-scale QS-21 purification, but to meet increasing demand, clonal plantation forestry will be the most practical and scalable strategy in the near future. Alternative approaches, including plant cell culture, engineered microorganisms, and chemical synthesis, remain scientifically promising but currently face technical and economic limitations for large-scale pharmaceutical production.

## Introduction

**Figure 1:**
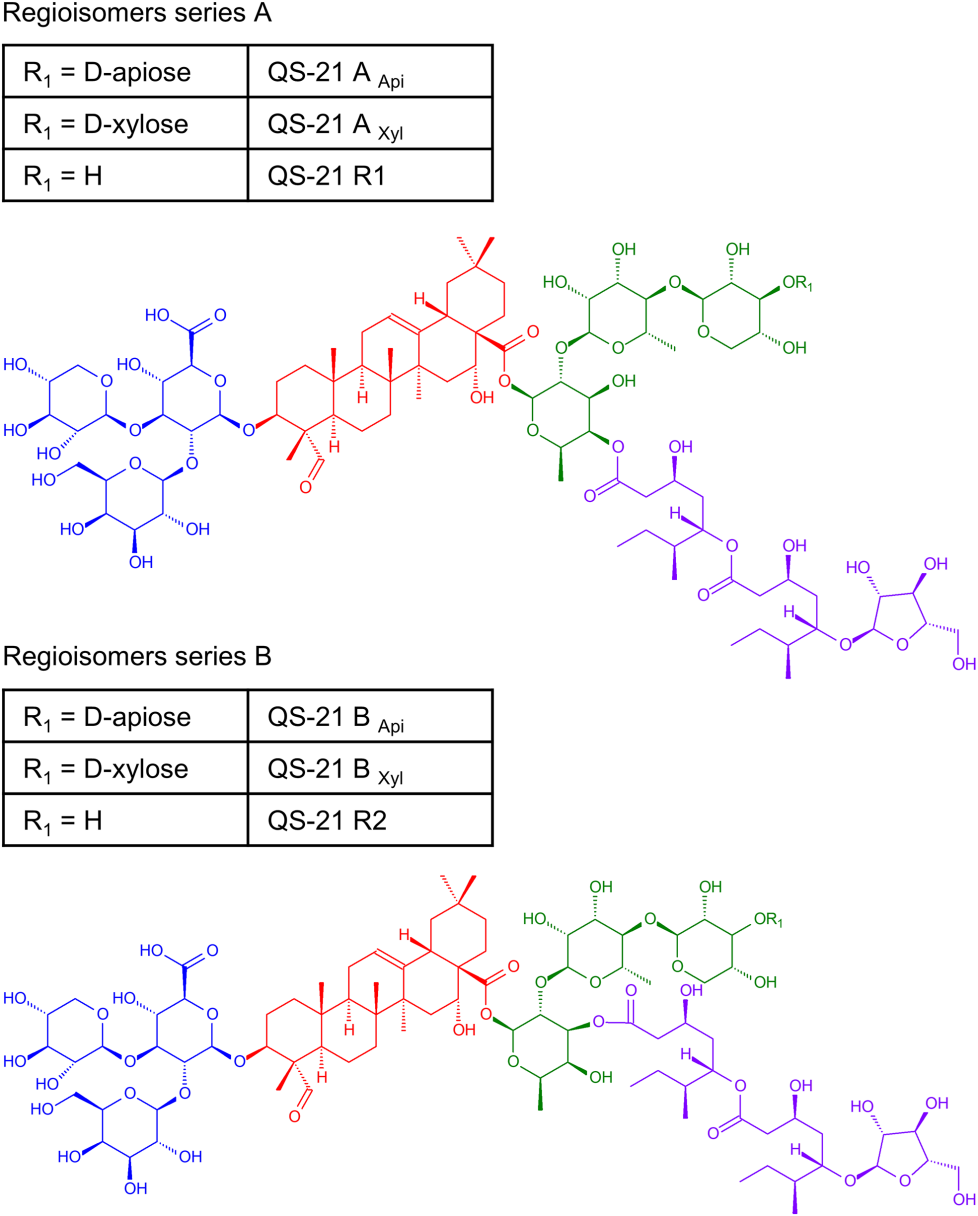
Chemical structure of QS-21 isomers QS-21_Api_ A/B, QS-21_Xyl_ A/B and minor derivatives QS- 21 R1 and QS-21 R2. Each saponin component contains a central quillaic acid triterpene core (red), a branched trisaccharide (blue), a linear tri/tetrasaccharide (green) and an arabinosylated fatty acyl chain (violet).

Adjuvants are components of vaccines increasing their efficacy by stimulation and boosting of the immune response (1). Although adjuvants play a critical role in modern vaccinology, the number of adjuvant systems approved for human use remains limited; the U.S. Food and Drug Administration (FDA) has approved only six adjuvants for use in human vaccines, a list whose components include aluminum salts, monophosphoryl lipid A, cytosine phosphoguanine, squalene and the fractions of triterpenic glycosides QHA, QHC and QS-21 from *Quillaja saponaria* tree (2, 3). QS-21 (**Figure 1**) has been tested and validated in over 120 clinical trials (1).

Today, the commercial production of QS-21 relies only on their extraction of the bark of *Q. saponaria* collected in wild Chilean forests. The severe ecological damage in native forest resulting from the unregulated exploitation of the bark *Q. saponaria* before the 1990s (4), as well as the variability in molecular composition of saponins seen even among *Q. saponaria* trees of similar age and local environment (5) have motivated some concerns about the availability and reliability of natural sources of QS-21 to cope with the expanding global use of saponin-based vaccine adjuvants (6).

In this context, the present review summarizes the historical development and chemical characteristics of QS-21 and examines current and emerging production platforms with emphasis on scalability, consistency, and sustainability.

## History of the use of triterpenic saponins from Quillaja tree

**Figure 2:**
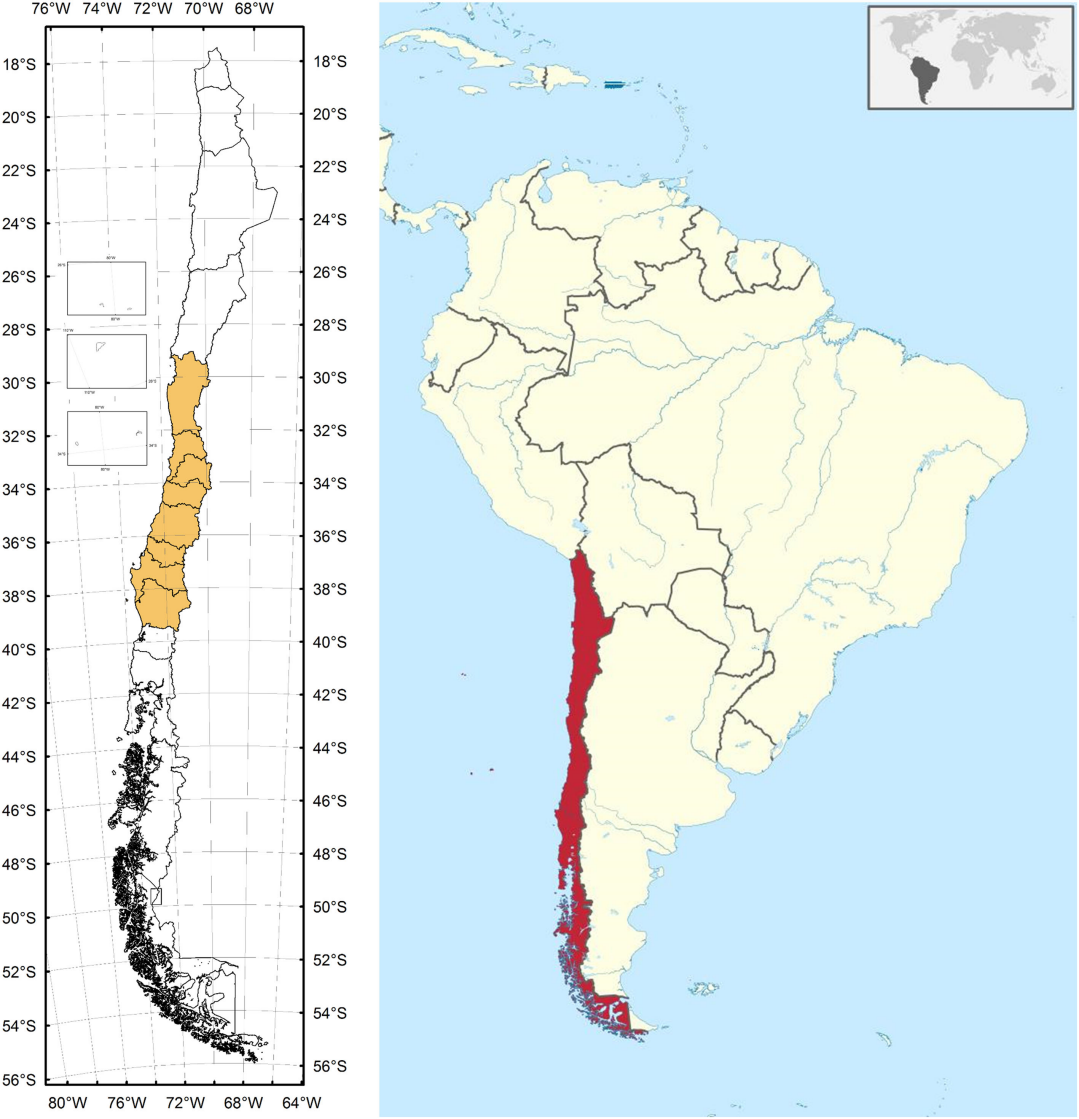
*Q. saponaria* grows in a wide range of soil conditions along the Coastal Range and the foothills of the Andes Range in semiarid central Chile with longitudinal distribution from 30° to 38° S. In the map of Chile (left panel) the regions where *Q. saponaria* grows are highlighted. Source of *Q. saponaria* distribution, https://catalogoplantas.udec.cl/?q=node/5045; source of map of Chile in South America, https://es.wikipedia.org/wiki/Archivo:Chile_in_South_America.svg.

The native Chilean tree *Quillaja saponaria* Molina (known as ‘quillay’ in Chile) is an evergreen, medium-sized tree that can grow up to 15 m in the wild forests of Central Chile (7), where it thrives along various shrubby species forming the Chilean Mediterranean scrub (8). *Q. saponaria* grows in a wide range of soil conditions along the Coastal Range and the foothills of the Andes Range in semiarid central Chile with longitudinal distribution from 30° to 38° S (**Figure 2**); the altitude of quillaja forests ranges from 15 m in the coastal hills to 1, 750 m in the Andes, covering precipitation ranges from 200 to over 1, 000 mm/year (8–10).

Since ancient times, the inner bark of the tree has been widely used to prepare homemade soap (7). During the first half of the 19^th^ century, this tensoactive property of the quillaja extracts led to propose industrial methods for their preparation by alcoholic extraction of the bark, as well as the use of such extracts as ingredients for medicinal formulations (11, 12). Le Beuf specifically suggested the use of purified quillaja extract as an effective emulsifier for the preparation of medicinal formulations – e.g. quinine and morphine.

**Figure 3:**
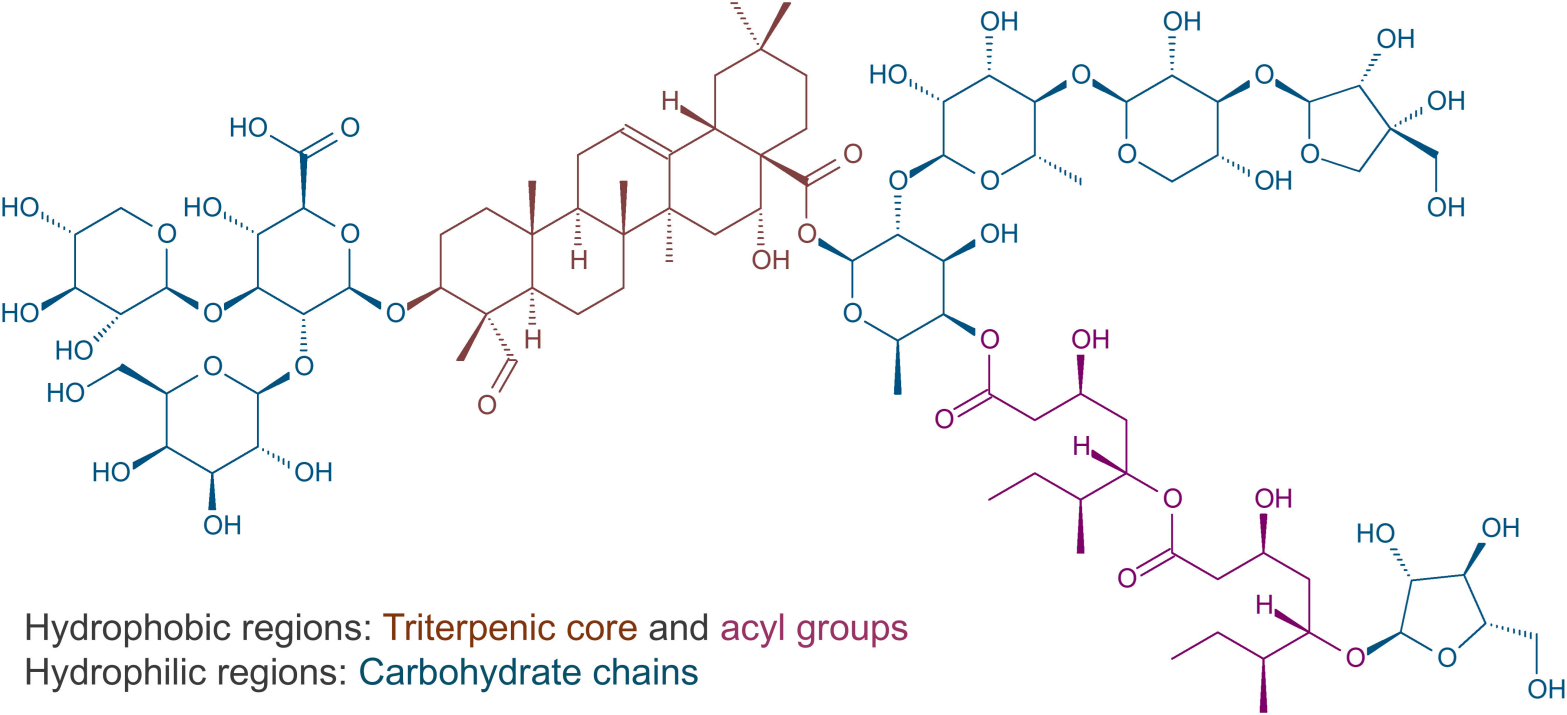
*Q. saponaria* saponins are surfactants (surface active agent), amphipathic molecules with hydrophilic and hydrophobic regions. The figure highlights both regions in the structure of QS-21_Api_.

The compounds behind the tensoactive properties of quillaja extracts are saponins, a class of high-molecular weight glycosides widely spread in the plant kingdom (13). Structurally, quillaja saponins are amphiphilic compounds composed of two hydrophilic tails (composed mostly by sugars) and a lipophilic triterpenic part (the aglycone quillaic acid QA) and a partially lipophilic arabynosylated fatty acyl chain (14–17) (**Figure 3**). The amphiphilic nature of quillaja saponins determines their surface activity (e.g. their ability to form stable foams and act as emulsifying agents) (13). The biomass of *Q. saponaria*, particularly the inner bark, is an abundant source of those triterpenic saponins (7); although QA is the most abundant saponin aglycone in *Q. saponaria*, there are few quillaja saponins derived from 22-β-hydroxyquillaic acid, phytolaccinic acid, 23-*O*-acetylphytolaccinic acid and echinocystic acid reported in the literature (18). Quillaja saponins – as well as the aglycone QA – also have shown multiple biological activities: anti-inflammatory, anti-microbial hemolytic, antinociceptive and cytotoxic (19–21). However, the most impressive effect of saponins is the immunostimulant effect, which led over the years to the development of Quillaja-derived saponins as vaccine adjuvants (22).

## Early observations of saponin immunopotentiation by quillaja extracts and the discovery of the active principle

The outstanding properties of saponins as immunostimulant agents for vaccine formulation were discovered after the work of Gaston Ramon (23, 24) who theorized that the addition of non-antigenic substances (e.g. tapioca powder) to antigens increased its value of specific immunity. Ramon successfully improved the immune response of horses against attenuated diphtheria toxin by mixing with tapioca powder (24). Following that example, Galea & Tzortzakis (25) injected emulsions of aphthovirus (provoking foot and mouth disease, FMD) with quillaja extract to healthy guinea pigs, preventing further development of the disease (controls injected with virus without saponin developed the disease after 36–60 h).

Although a commercial vaccine against FMD formulated with quillaja extract was introduced in Argentina for the immunization of cattle (26), it was not until the 1970s that saponins were identified as the immunostimulant agents in quillaja extracts (27). During the late 1980s, Kensil et al. (28) separated 22 chromatographic fractions of quillaja saponins from crude bark extracts by reverse phase HPLC; since then, the structure of more than 50 saponins of *Q. saponaria* have been described (18).

**Figure 4:**
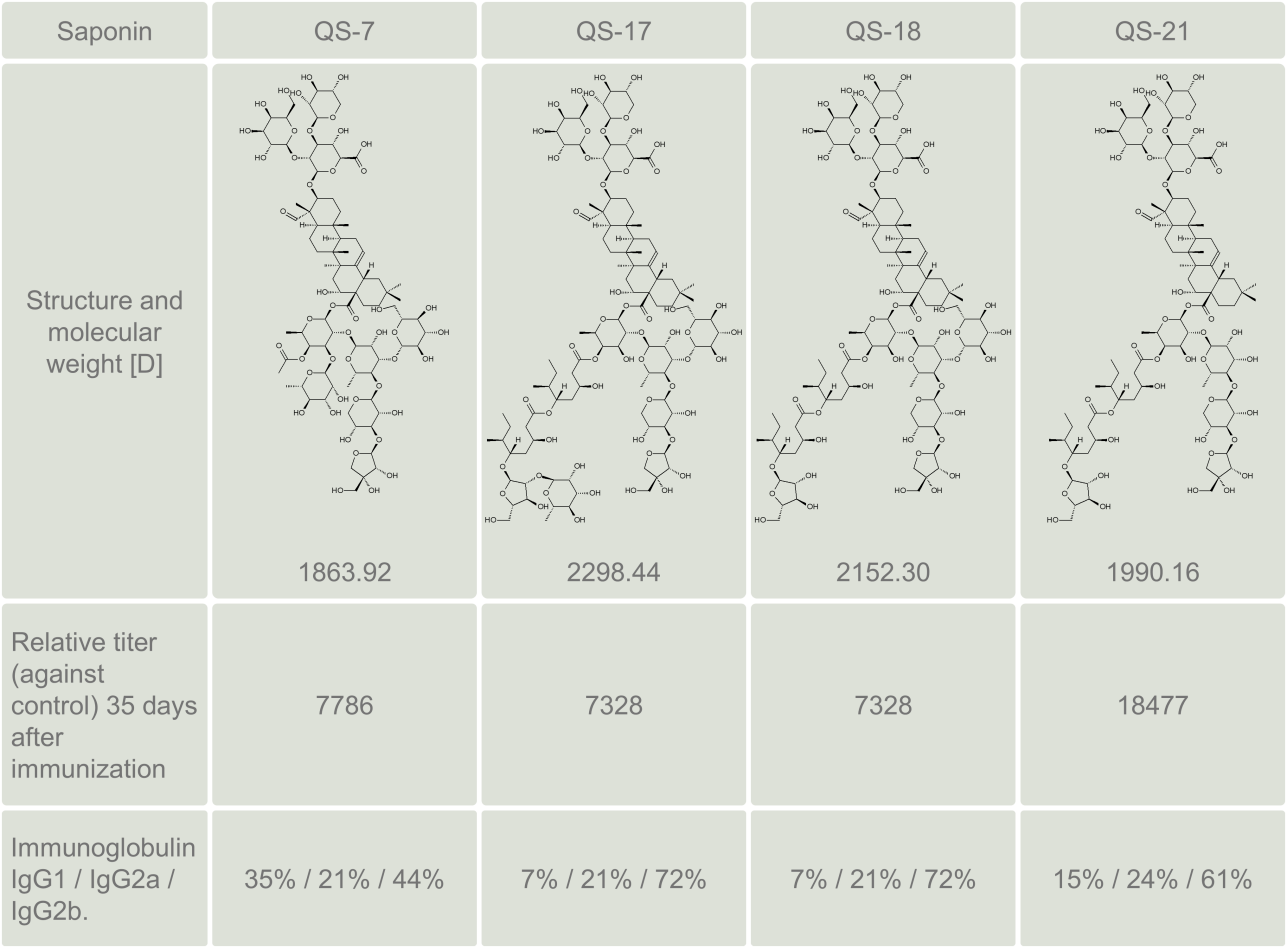
Summary of structure, molecular weight and immunostimulant properties of the most abundant saponin components QS-7, QS-17, QS-18 and QS-21, according to Kensil et al. (22, 28). The immunostimulant properties were tested in CD-1 mice with 20 μg cyt b5 (antigen) and 20 μg of each saponin. The figure shows the relative IgG titer against control without adjuvant.

**Figure 5:**
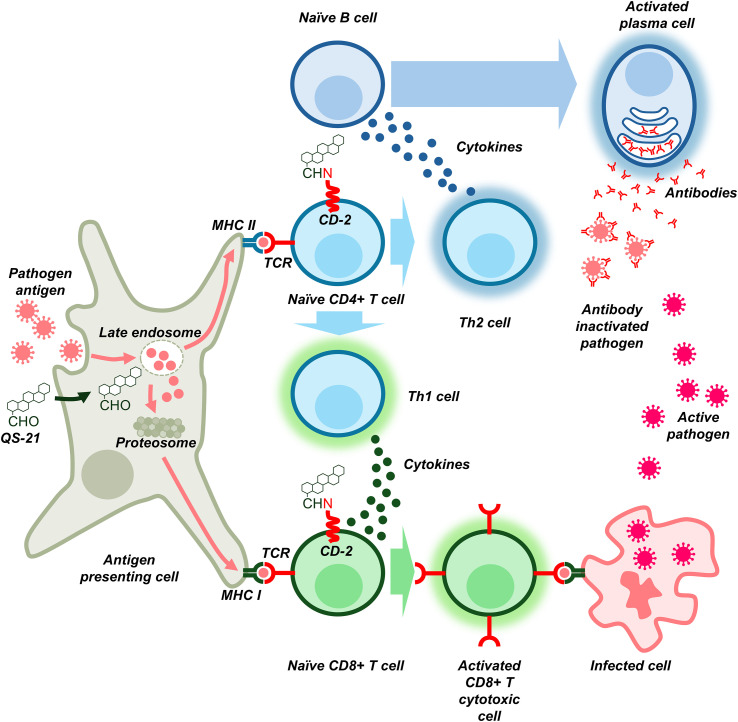
Simplified diagram of the mechanism of action of QS-21. This saponin acts at two levels, at antigen presenting cells (e.g. dendritic cells) and in naïve T lymphocytes. Antigen presenting cells engulf the antigen and partially degrade it in the endosome before presentation to naïve CD4+ T cells as MHC II- antigen complexes binding the T-cell receptors TCR; this activates CD4+ T to Th1 and Th2 cells. Th2 cells lead to humoral immunity via activation of B cells to antibody producing plasma cells. QS-21 partially disrupts endosome membrane of the antigen presenting cell, allowing the escape of partially degraded antigen to the proteosome to be further presented on cell surface as a complex MHC I-antigen. This complex can activate naïve CD8+ T cells, which become the active cytotoxic cells able to detect and destroy pathogen infected cells; this activation process is costimulated by cytokines produced by Th1 cells. In a second level, the aldehyde group of QS-21 can attach covalently to amino groups of T-cells receptors (most likely ϵ amino group of lysine residues in CD-2 protein), delivering a costimulatory signal to T cells that enhances the activation by antigen presenting cells. To simplify the diagram, other protein-receptor interactions between lymphocytes and antigen presenting cell are not shown.

In a seminal study, Kensil et al. (22) characterized the immunostimulant properties of the most abundant saponin components QS-7, QS-17, QS-18 and QS-21 (**Figure 4**). Although the main saponin component QS-18 was found to be highly toxic in mice, that study demonstrated that QS-7 and QS-21 were far less toxic and comparable in terms of immunostimulant activity. However, QS-21, being more abundant than QS-7 in *Q. saponaria* bark, has been the most widely studied saponin adjuvant for more than 30 years since the Kensil’s study (29). Subsequent research demonstrated that QS-21 also boosts the CD8+ CTL response to subunit antigens (30). **Figure 5** summarizes the mechanism of action of QS-21.

## QS-21 takes the spotlight: a new era of saponin adjuvants

### Decoding QS-21: structure, isomers, and nomenclature

The name “QS-21” initially referred to the 21^st^ chromatography fraction reported by Kensil after the fractionation of *Q. saponaria* bark reversed-phase HPLC chromatography (28). Subsequent chromatographic and spectroscopic studies (greatly helped by the previous structure elucidation of the larger saponin QS-17 (31–33) led to the identification of four isomeric forms of an acylated triterpene glycoside with a molecular formula of C_92_O_46_H_148_ and a molecular weight of 1, 990 Da (34, 35). The four isomers consist of a central quillaic acid triterpene core, flanked on either side by a branched trisaccharide (bound to C3 position of quillaic acid) and a linear tetrasaccharide (bound to C28 position), which is in turn connected to an arabinosylated fatty acyl chain. That chain is bonded to a D-fucose residue of the tetrasaccharide; however, intramolecular trans- esterification of this bond between the 4- and 3-hydroxyl groups on the fucose ring occurs naturally in solution, resulting in two regioisomers in equilibrium, QS-21A and QS-21B, respectively (34, 36). The terminal sugar of the tetrasaccharide is either D-apiose or D-xylose, giving rise to two compositional isomers denoted as QS-21_Api_ and QS-21_Xyl_ with comparable adjuvant activity (35). Later it was found that QS-21 may contain additional minor derivatives QS- 21 R1 and QS-21 R2 lacking the pentose at the end of the tetrasaccharide bound to the C-28 position of quillaic acid (37). The structures of the components of QS-21 are shown in **Figure 1**.

### QS-21 in licensed and investigational vaccine formulations

Saponins have been incorporated into multiple adjuvant systems differing in composition and structure. These systems range from formulations containing a single immunostimulatory saponin component to more complex liposomal or nanoparticle assemblies combining saponins with additional immune activators. saponin-containing formulations span licensed vaccines and candidates in clinical development (38). While some platforms remain at preclinical or early-phase stages, ongoing translational efforts suggest continued diversification of saponin-based adjuvant technologies.

Among simpler systems containing a single saponin-based immunostimulant are oil-in-water emulsions such as AS02 (GSK) (39) and SQ (Vaccine Formulation Institute) as well as the SQ liposomal formulation also developed by VFI (40). These formulations have been evaluated in pre-clinical and early clinical studies.

More complex formulations combine QS-21 with the TLR4-agonist 3-O-desacyl-4′-monophosphoryl lipid A (MPLA). The most widely implemented example is AS01 (GSK), a liposomal MPLA+QS-21 system incorporated into licensed vaccines against herpes zoster, malaria, and respiratory syncytial virus (41–46). A related MPLA+QS-21 liposomal formulation, ALFQ, has been developed by the U.S. Walter Reed Army Institute of Research (WRAIR) and recently evaluated in multiple clinical studies (47). Additional liposomal MPLA+saponin platforms include SPA14 (Sanofi) (48), BFA01 (RecBio) (49), and LMQ/SMQ systems developed by VFI (40).

Nanoparticle-based systems incorporating *Quillaja* saponins represent another structural class. Matrix-M (Novavax), composed of saponin-based nanoparticles enriched in QHA and QHC saponin fractions (50), is included in licensed and investigational vaccines (51). Another nanoparticle platform, SMNP, combines saponins and MPLA within nanoparticulate assemblies and has been evaluated in pre-clinical studies (52, 53).

### Supply capacity and sustainability considerations

The continued development and approval of vaccines incorporating QS-21 have implications for supply capacity. Export data from Chile indicate a substantial increase in the commercial value of purified saponin extracts between 2015–2019 and 2020–2024 (54). While these figures include multiple saponin products and reflect broader market dynamics, they illustrate the expanding industrial relevance of Quillaja-derived extracts.

There is a limited number of suppliers of quillaja extracts and QS-21 for use in vaccine industry, with an overall estimated production capacity of ~ 10 Kg/year of GMP QS-21; that amount would suffice to produce 200 million doses of malaria vaccine Mosquirix (containing 50 µg of QS-21) or 400 million doses of the new tuberculosis vaccine M72/AS01_E_ (containing 25 µg of QS-21). There are five major suppliers of QS-21 (3, 55):

- Desert King: A company with manufacturing facilities in Chile. Currently, Desert King is a supplier of quillaja extracts and QS-21 for approved human vaccines The production capacity of QS-21 is 1 Kg/year; the capacity is expected to increase to 5 Kg/year in the near future, and later to 10 Kg/year (3).
- Q-Vant: Another company with production facilities in Chile; is a recent market entrant whose production capacity of QS-21 is estimated to be 1 Kg/year (3).
- Botanical Solutions Inc: Another recent market entrant; a company with roots in Chile and operations in California, claiming the development of a proprietary tissue culture platform to produce QS-21. There is no data to estimate their current production capacity, although according to company’s report they would produce a few Kg/year of QS-21 (3).
- AmerStem: A California based company using aeroponics, vertical farming and botanical methods to produce raw material for the purification of QS-21. AmerStem would produce 2 Kg/year in the near term (3).
- SaponiQx (an Agenus subsidiary): During April 2024, announced the availability of Stimulon™ cultured plant cell QS-21 (cpc QS-21) on the international retail infrastructure InvivoGen - a company established in 1997 in San Diego (55).

## Technology for production of QS-21: traditional and alternative raw materials

The general approach for purification of QS-21 involves several steps:

- Production of a suitable source of raw material, e.g. bark collected in Chilean wild forests (currently employed by Desert King), clone biomass from selected chemotypes grown in plantations (in prototype stage by Desert King) (56), cell biomass produced by liquid culture in bioreactor of either *Q. saponaria* callus cells, described by SaponiQx (38) as well as engineered *S. cerevisiae* expressing the biosynthetic genes of QS-21 pathway (57). Other raw material sources are cited in the literature; Amerstem claims the production of *Q. saponaria* plantlets by vertical farming (3), Botanical Solutions Inc. (BSI) is using indoor culture platform to produce *Q. saponaria* plant tissue (58). Unfortunately, no published data is available of the technical details and yields of the latter approaches.
- The extraction of the saponins from the raw material, followed by the removal of non-saponin components in the extract to obtain a purified saponin extract (>90% w/w on dry solids). According to Resnik et al. (59), by treating quillaja biomass with hot water (70-80 °C), an extract with a saponin content in the extracted solids of about 20% can be obtained. The crude extract is treated with decolorating agents (PVPP), followed by filtration over diatomaceous earth, to remove colored impurities that tend to precipitate during storage (e.g. protein-polyphenol complexes). The product is further purified by selective precipitation and ultrafiltration to remove most non-saponin components such as polysaccharides, polyphenols, sugars, inorganics, that may interfere in the purification of QS-21 by chromatography followed by pasteurization and spray drying, rendering extracts with saponin purity ≥ 70 w/w (59, 60). The same basic approach (without ultrafiltration step) was described by Baig et al. (61, 62) for the production of a mid-purity bark extract suitable for further chromatographic isolation of QS-21. With modifications, the above approach could be adapted to the production of saponin rich extract from cell material produced in bioreactors.
- The chromatographic fractionation to isolate QS-21 saponin may be performed combining several technologies, such as normal phase chromatography on silica gel, reverse phase chromatography and/or hydrophilic interaction chromatography (HILIC); after collection of the QS-21 fractions, solvents can be removed by low-pressure evaporation followed by freeze-drying (22, 28, 61–64).

Chemical synthesis of QS-21 deviates of the above general approach, although the chromatographic purification is mandatory at the end of such process; it must be kept in mind that the starting material for that process is quillaic acid produced from an extract of quillaja biomass (65, 66).

In the following paragraphs the different raw material sources and purification technologies are described and compared in terms of their feasibility for large scale production of QS-21 - **Table 1** provides a summary of the main features of each method along the estimated requirements of raw materials to produce 50 Kg/year (equivalent to 1, 000, 000, 000 50 µg doses, or 2, 000, 000, 000 25 µg doses). All estimates are based on publicly available data and simplified assumptions. The results should be interpreted as order-of-magnitude estimates intended for comparative analysis rather than precise industrial predictions. A supplementary file is provided with detailed calculations of the yields for each production technology.

**Table 1:** Summary of available technologies for production of QS-21; comparison for production of 50 Kg of QS-21 per year (1, 000, 000, 000 50 µg doses/year, or 2, 000, 000, 000 25 µg doses/year).

Technology.	Purification from extracts of selected Quillaja bark collected in the wild forest.	Purification from extracts of bark harvested from clonal quillaja trees grown in plantations.	Purification from extracts of aerial biomass harvested from clonal quillaja trees grown in ultra high density plantations.	*De novo* synthesis of QS-21 in cultures of *Q. saponaria* cells.	*De novo* synthesis of QS-21 in cultures of *S. cerevisiae* cells engineered with genes involved in its biosynthesis.	Chemical synthesis of QS-21: Glycosylation/acylation of quillaic acid from natural sources.
Process type.	Production of a highly purified saponin extract followed by chromatographic isolation of QS-21.	Operation of tree plantations as source of raw material. Production of a highly purified saponin extract followed by chromatographic isolation of QS-21.	Operation of tree plantations as source of raw material. Production of a highly purified saponin extract followed by chromatographic isolation of QS-21.	Operation of bioreactors to produce plant cell cultures as source of raw material. Production of a highly purified saponin extract followed by chromatographic isolation of QS-21.	Operation of bioreactors to produce yeast cultures as source of raw material. Production of a highly purified saponin extract followed by chromatographic isolation of QS-21.	Chemical synthesis of QS-21 from quillaic acid produced by hydrolysis of a quillaja extract Type 2 produced from *Q. saponaria* biomass (wood + bark).
Company.	Desert King.	Desert King.	Desert King.	SaponiQx.	Not publicly disclosed.	Not publicly disclosed.
Status	Operational.	Prototype plantation is currently on growing stage. First batch of extract scheduled to 2033.	Prototype plantation is currently on growing stage. First batch of extract scheduled to 2027.	Operational.	Not publicly disclosed.	Not publicly disclosed.
Raw material from quillaja.	Selected bark.	Bark from clonal trees.	Aerial biomass from clonal trees.	Callus cells from selected *Q. saponaria* trees grown in bioreactors.	Yeast cells engineered with genes involved in the biosynthesis of QS-21, grown on bioreactors using glucose, galactose and 2-methylbutiric acid as substrate.	Quillaja extract Type 2 produced from *Q. saponaria* biomass (extraction, followed by clarification, ultrafiltration and spray-drying).
Raw material requirement for 50 Kg QS-21/year.	200 Ton/year of dry bark previous to the selection.	100 Ton/year of dry bark.	374 Ton/year of dry biomass.	54, 200 m^3^/year (recovery of 923 μg/L). *Note: Estimation based on data reported by Lv et al.* (38)*. No data about reactor operation was provided by the authors; it is assumed that batch cycles elapse 82 h.*	528, 541 m^3^/year (recovery 95 μg/L) for QS-21_Xyl_; 1, 610, 000 m^3^/year (recovery 31 μg/L) for QS-21_Api_. *Note: Estimation based on data reported by Liu et al.* (57)*. The authors report four days operation; it is assumed that batch cycles elapse 106 h (96 h + 10h for cleaning and sterilization).*	33 Ton/year of Type 2 extract (produced from 1, 970 Ton/year of dry biomass from adult trees).
Other requirements.	Plant capacity to process the bark.	Cloning capacity of 16, 000 plants per year. 16 hectares of clonal trees grown 15 years (plant density; 1, 000 trees/hectare). For continuous supply of raw material, 15 land pads (16 hectare each) must be operated (total planted surface; 240 hectares).	Cloning capacity of 1, 100, 000 plants per year. 22 hectares of clonal trees grown 3 years (plant density; 50, 000 trees/hectare). For continuous supply of raw material, 3 land pads (22 hectare each) must be operated (total planted surface; 66 hectares).	Ancillary equipment for production of inoculum for bioreactor operation. Large bioreactor capacity. The world’s largest plant cell culture cGMP facility (situated in Ahrensburg, Germany) is used to produce paclitaxel on a 75, 000 L scale (86); assuming an operational cycle of 82 h per batch, at least 7 bioreactors of 75 m^3^ would be required operating in a non-stop mode throughout a year.	Ancillary equipment for production of inoculum for bioreactor operation. Large bioreactor capacity. Assuming an operational cycle of 106 h per batch (96 h operation + 10 hours cleaning and sterilization), at least 30 bioreactors of 215 m^3^ would be required operating in a non-stop mode throughout a year to produce QS-21_Xyl_ (the production of the isomer QS-21_Api_ would require three-fold plant capacity).	Chemical synthesis facility large enough to process raw material.
CO_2_ fixation associated to the quillaja raw material collected in a year.	3, 300 Ton of CO_2_.	1, 670 Ton of CO_2_.	910 Ton of CO_2_.	The process does not operate in photosynthetic mode; no CO_2_ is fixed during the process.	The process does not operate in photosynthetic mode; no CO_2_ is fixed during the process.	3, 600 Ton of CO_2_.
Scalability to 50 Kg QS-21/year.	Moderate	High	High	Low	Low	Low
Advantages	Wild forest management promotes growth and preservation.	The land requirement is rather modest. Once bark is harvested, the trees are ready for a new growth cycle of 15 years. Since the raw material is bark, the current processing methods are fully applicable. Selected clones are already available and patented.	Once biomass is harvested, the trees are ready for a new growth cycle of 3 years. Selected clones are already available and patented.	It allows the production of QS-21 in any place in the world. Production of less complex saponin profiles may reduce fractionation costs.	It allows the production of QS-21 in any place in the world. Depending on the genes integrated in *S. cerevisiae* genome, the isomers QS-21_Api_ and QS-21_Xyl_ could be produced pure.	It allows the production of QS-21 in any place in the world, provided that Type 2 extract is available. The isomers QS-21_Api_ and QS-2 _Xyl_ can be produced pure.
Challenges	200 Ton/year of dry bark would be the practical sustainable limit for wild forest sources in Chilean forest. The surplus of collected bark must be employed in the production of other adjuvants.	Time involved in the initial growth of the trees before productive stage (15 years).	To this size of operation, the cloning capacity must be pushed to the limit. The processing of the raw material requires some changes in the traditional processing method.	The bioreactor capacity required is huge. The operation of plant cell culture is extremely sensitive; extreme care is required for the operation of large bioreactors in order to prevent contamination events and mechanical cell damage. The concentration of QS-21 in the yeast culture is very low; the costs of downstream processing might be high.	Extreme care is required for the operation of large bioreactors in order to prevent contamination events. Is not clear the genetic stability of a yeast strain harboring a large number of heterologous genes; extreme care must be applied to keep a backup of the yeast strain. The concentration of QS-21 in the yeast culture is very low; the costs of downstream processing might be high.	Mostly related to the yields; after each synthetic step some purification is required.

These estimates are theoretical extrapolations based on reported titers and assume ideal recovery efficiencies. Actual industrial performance may vary.

### Traditional process: wild forest as a source of raw material for production of QS-21

The traditional production of QS-21 involves the harvesting and selection of the quillaja bark collected from wild forests (4). For harvesting, the external part of the bark (cork cambium layer) is removed with knives, followed by the removal of inner bark used for saponin production (59). The selection is a mandatory step for bark harvested from wild forests; this step is crucial aiming to a homogeneous and stable raw material fulfilling the requirements for the specific product and application. The selection criteria arexmostly based in the analysis of bark extracts by liquid chromatography.

Parameters such as the fractional content of QS-21 in the total saponins as well as the content of saponins eluting close to QS-21 during the chromatographic purification determine the best available lots of bark for the process (5, 56, 61, 62). The lots of bark unsuitable for production of pure QS-21 are not discarded but used in the production of saponin fractions employed as ingredients of other vaccine adjuvants (suitable for human as well as for veterinary use).

It is estimated that the maximum sustainable amount of harvestable bark is ~ 600 Ton/year (3); since the chemical composition of trees in wild forest are not homogeneous, and considering that sampling and analysis of each tree in the forests is impossible, about 50% of the bark purchased in the field might be suitable as raw material for extracts intended as starting materials for the purification of QS-21.

That bark is sequentially processed to produce a high purity powder extract containing ≥ 90% w/w total saponins (60) followed by its chromatographic fractionation to obtain QS-21. Using that process, ~ 50 Kg/year of QS-21 would be isolated from extracts produced from 100 Ton/year of selected bark of *Q. saponaria*; that figure is estimated more than enough to fulfill the current estimated demand of QS-21 by pharmaceutical industry. As mentioned above, the surplus of bark purchased in this scenario (100 Ton/year) can be employed in the manufacturing of other precursors of adjuvants. It must be mentioned that the production of 200 Ton/year of dry bark in the forest is associated to the fixation of ~3, 300 Ton CO_2_, assuming that bark is the 11% of the dry biomass produced by the tree (67) and the content of carbon dry tree biomass; ~ 50% w/w (68).

A major concern related to the wild forest as source of raw materials for the production of quillaja extracts is the chemical homogeneity of the raw material. As mentioned previously, to purify the immunostimulant QS-21 by chromatography, it is highly desirable to have a homogeneous and consistent raw material with only minor amounts of other types of saponins eluting close to QS-21. Obtaining large amounts of Quillaja biomass with a homogeneous saponin composition is technically difficult because of the chemical variability between trees growing in the wild forest. Although the selection of specific trees for harvesting in the wild forest would in principle allow the collection of homogeneous materials (5), its practical implementation is rather difficult since it requires laborious procedures of sampling and chromatographic analysis of individual wild trees before harvesting of homogeneous Quillaja bark suitable as raw material.

### Plantations of clones of selected quillaja trees as a source of raw material for production of QS-21

**Figure 6:**
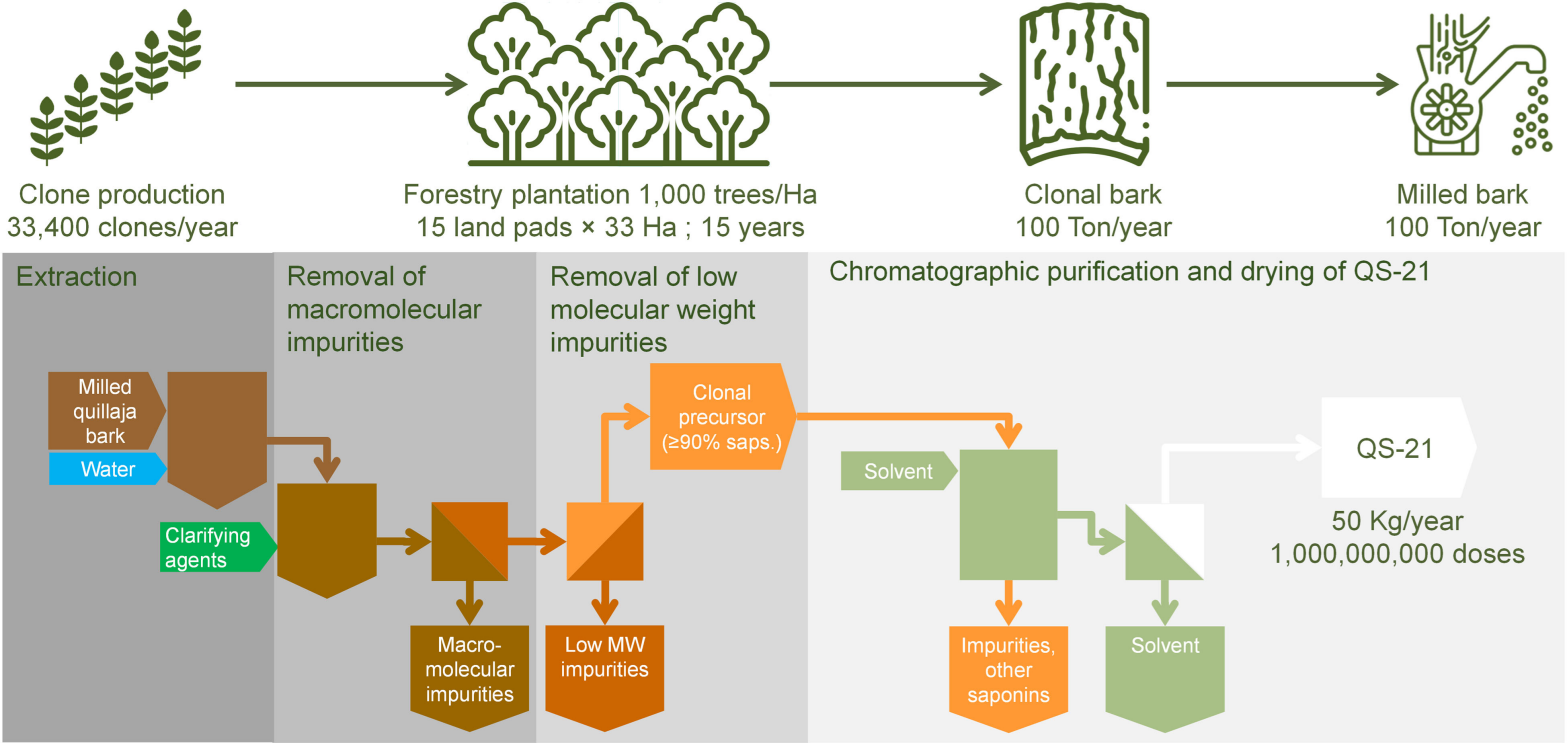
Diagram of the purification process of QS-21, using bark from clone trees grown 15 years in a forestry plantation (1, 000 trees per hectare).

**Figure 7:**
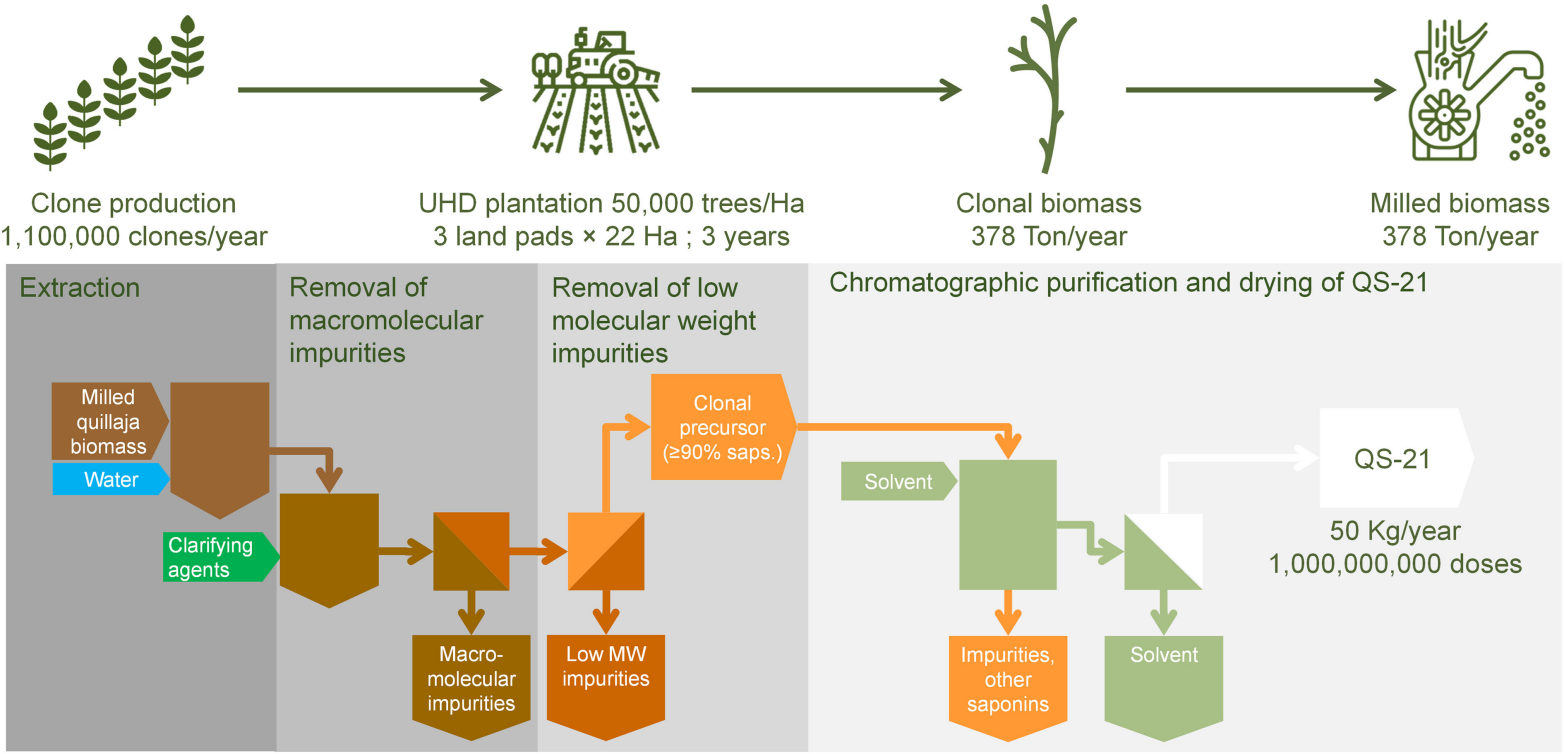
Diagram of the purification process of QS-21, using biomass from clone trees grown 3 years in a ultra-high density plantation (50, 000 trees per hectare).

To overcome the above concern related to the use of raw materials from the wild forest, the plantation approach arises as a promising and innovative way to produce at large scale homogeneous quillaja biomass for the production of QS-21 and other saponins (56). Desert King has pioneered the efforts to domesticate *Q. saponaria* and has developed plantation technology and selection methods of chemotypes suitable for the production of specific types of saponins (56); currently two new varieties of *Q. saponaria* suitable for QS-21 production are patented (69, 70). Two approaches are in prototype stage at Desert King: Forestry plantations and Ultra-High-Density plantations (UHD):

- Forestry plantations: This technology is based on plantations of clone trees of suitable chemotype at a density of 1, 000 plants/hectare. Under the weather conditions in the central area of Chile, quillaja trees are expected to be suitable for harvesting after 15 years without requirements of fertilization or irrigation; upon harvesting the trees are able to regrow and produce more biomass in a cycle of 15 years. A total surface of just 240 hectares (divided in 15 sectors of equal surface; only one sector being harvested per year; **Figure 6**) would produce 100 Ton/year of bark rendering after processing 50 Kg of QS-21 with the currently available technology. The advantages of this approach are the small number of clones required in the initial phase of the operation (only 240, 000 plants) and the production of a raw material equivalent to the current selected bark. The raw material produced is ready to use (since the chemotype is suitable by design of the plantation) and no need for laborious sampling and analysis of bark is required previous to the harvest. Since the initial stage of growth of the trees before the productive stage is very long (15 years), this approach requires careful planning considering the future demand of the product. Currently Desert King is operating a prototype plantation of this type (total surface 15 hectares) which will be entering to productive stage by 2033. As mentioned previously, the land size and the number of clone plants involved in the production of raw material for 50 Kg/year of QS-21 are relatively small, allowing further scaling up to fit with even larger demands in the future. Additionally, the production of 100 Ton/year of bark is linked to the fixation of ~1, 670 Ton CO_2_. A careful planning of the plantation location allows to mitigate the risks of wildfires and the optimization of harvesting/transport cost of the raw material to the production plant. On the other hand, quillaja plantations might be an attractive alternative to landowners currently producing other tree or plant species. Last but not least, this type of operation has no detrimental impact on native forests.
- UHD plantations: This technology is closely related to forestry plantations; it is based on plantations of clone trees of suitable chemotype at a density of 50, 000 plants/hectare. Such high density allows growth cycles of at most 5 years. Although juvenile plants are not a suitable source of bark, research on plantation technology at Desert King revealed that aerial biomass of juvenile plants of 3 years are suitable sources of QS-21 (56). In order to secure proper growth in a short cultivation time, fertilization and irrigation is required. Similarly to forestry plantations, the plants are able to regrow after harvest of the aerial biomass in a cycle of 3 years. At least three sectors must be operated in parallel in shifted fashion in order to have at least one sector ready to harvest every year. A total surface of 66 hectares (3 sectors of 22 hectares each; **Figure 7**) would produce 374 Ton/year of dry aerial biomass rendering after processing 50 Kg of QS-21. The advantages of this approach are the short operational times compared to forestry plantations. Also, the harvesting of juvenile biomass can be easily mechanized compared to the bark recollection from trees in forestry plantations. Again, the raw material produced is ready to use and no need for sampling and analysis of bark is required before the harvest. However, the clone propagation for the initial stage is quite demanding, although feasible (1, 100, 000 clones/year). Currently Desert King is operating a prototype plantation of this type (total surface 1.5 hectares) which will be entering to productive stage by 2027. The production of 374 Ton/year of biomass is linked to the fixation of ~910 Ton CO_2_. Other advantages previously mentioned in the discussion of forestry plantations also apply to UHD plantations (mitigation of risks of wildfires, optimization of harvesting/transport cost of the raw material to the production plant, use as alternative crop for landowners and reduced impact on the native forest).

### Production of QS-21 by *de novo* synthesis in cultures of *Q. saponaria* cells

An innovative process to produce saponins is based in cultured cells of *Q. saponaria* in liquid medium, where the cells are originated from a callus tissue culture (71). Lv et al. (38) describe the testing of 23 mixed cell cultures created from cuttings taken from matures quillaja trees sampled in Chile; the authors found 14 cultures were able to produce QS-21 (concentrations in the mg/L range). Particularly interesting is the similarity of the chromatographic profiles of extracts from bark and plant cell culture. Starting from 26 L of liquid culture those authors were able to purify 24 mg of QS-21 by reverse phase chromatography (the identity of the product was confirmed by mass spectroscopy and ^1^H and ^13^C NMR spectroscopy). The same authors verified the biological activity of the isolated product against a control of QS-21 purified from quillaja bark; the results show comparable induction of immunity responses during *in vivo* testing.

Although the authors claim that this technology is scalable as a manufacturing process of QS-21 and related saponins, some care is required to validate that assert. Continuing with our comparison of the requirements involved in the production of 50 Kg/year of QS-21, we estimate a requirement of plant cell culture as high as ~54, 200 m^3^/year. That figure is monumental, and as will be discussed later in this review, it is not clear the feasibility of the production of QS-21 at large scale employing this technology.

### Production of QS-21 by *de novo* synthesis in cultures of engineered *Sacharomyces cerevisiae* cells

An even more innovative process was reported by Liu et al. (57) which is based in its production by liquid cultures of yeast cells engineered to synthetize all the enzymatic machinery involved in the synthesis of QS-21 in *Q. saponaria*. The biosynthesis of QS-21 can be dissected in the following components: i) the synthesis of quillaic acid aglycone; ii) the addition of activated sugar donors uridine-diphosphate sugars (UDP-sugars) required to the assembling of C3 tri-saccharide and C28 tetra-saccharide, and; iii) the synthesis and addition of the arabinosylated dimeric acyl unit to the fucose residue at the C28 tetra-saccharide. To achieve the production of QS-21 in yeast, the strain *S. cerevisiae* JWy601 with an upregulated mevalonate pathway was employed as a host for integration of genes involved in the synthesis of QS-21 – in this strain the genes of the enzymes involved in the conversion of acetyl-CoA to the terpene precursor farnesyl pyrophosphate are under control of galactose inducible promoters (72). In order to achieve the synthesis of QS-21from galactose and (*S*)-2-methylbutyric acid as substrates, genes of *Q. saponaria*, *Saponaria vaccaria* and *Arabidopsis thaliana* were integrated into the genome of JWy601 strain - under the control of galactose inducible promoters:

- Strain YL-46: 30 genes required for the biosynthesis of QS-21_Xyl_: 94.6 mg/m^3^ after 96 h of growth in liquid medium containing 40 Kg/m^3^ galactose and 500 g/m^3^ and (*S*)-2-methylbutyric acid.
- Strain YL-47: 32 genes required for the biosynthesis of QS-21_Api_: 31.1 mg/m^3^ after 96 h of growth in the same medium employed for YL-46 strain.

In a further refinement, the authors engineered yeast cells able to produce their own (*S*)-2-methylbutyric acid for QS-21 synthesis by inducible expression of a polyketide synthase from *Aspergillus terreus*; the yields of QS-21_Xyl_ or QS-21_Api_ obtained with those improved strains were not reported (57). Although the work of these authors is remarkable in terms of the strain engineering involved, the results are not easy to scale up for practical production of QS-21. Continuing with our comparison of technologies to produce 50 Kg QS-21/year, 528, 541 m^3^/year of culture would be required according to the data provided by the authors (we are assuming an unlikely 100% recovery of the saponin from the extract - the authors did not provide the actual yield from yeast culture). Such volume is huge, but still feasible for yeast fermentation, considering that industrial fed-batch production of baker’s yeast is performed in large bubble column bioreactors >100 m^3^ (73). One of the advantages is the selective production of the isomers QS-21_Xyl_ and QS-21_Api_ using cultures of a suitable strain.

### Chemical synthesis of QS-21

This method is based on the glycosylation/acylation of quillaic acid produced by hydrolysis of a quillaja extract Type 2 produced from *Q. saponaria* biomass. The production of 50 Kg/year of QS-21 would require 33 Ton/year of Type 2 extract (produced from 1, 970 Ton/year of dry biomass (wood + bark) from adult trees involving the fixation of ~3, 600 Ton of CO_2_).

The process starts with the purification of quillaic acid from a quilaja extract; according to Cartagena (74) this compound could be produced from a powder quillaja extract type 2 extract (containing 70% w/w on solid basis) by acid hydrolysis with HCl at reflux temperature, followed by silica gel and Sephadex chromatography (yield 0.95% w/w). As was noted by Kobert (75) and Windaus et al. (76), strong treatment with acid (reflux at 100 °C with HCl or H_2_SO_4_) led to complete hydrolysis of glycosidic and ester bonds joining sugar and fatty acid residues to the aglycone. However, under those harsh conditions, even the double bond in the free quillaic acid isomerized from Δ12 form to produce the more substituted (and more stable) Δ13 alkene (77); the above may explain the low yield of recovery of QA described by Cartagena (74).

Next, the quillaic acid is activated by carboxyl allylation with allyl bromide (yield 70% w/w) and next glycosylated at the 3-O position with a previously synthesized trisaccharide containing GlcA, Gal and Xyl, protected with benzyl ethers (BnO) groups (yield 59%w/w). Once the resultant intermediate is deallylated (yield 75% w/w) follows the addition to 28-O position of the BnO protected tetrasaccharide containing Fuc, Rha, Xyl, Api acylated with the arabinosylated dimeric acyl unit at the Fuc residue (yield 70% w/w). The resultant intermediate is deprotected (yield 75% w/w) rendering QS-21_Api_ (overall yield from quillaic acid, 0.154% w/w) (66). In a fairly similar approach (differing only in the addition of tetrasaccharide containing Fuc, Rha, Xyl, Xyl acylated with the arabinosylated dimeric acyl unit at the Fuc residue) QS-21_Xyl_ is obtained with an overall yield of 0.150% w/w from quillaic acid (65).

Although this method has some advantages such as the possibility to produce QS-21 in any place in the world (provided that Type 2 extract is available) and the independent preparation of pure isomers QS-21_Xyl_ and QS-21_Api_, it is not likely that this process replaces traditional approaches. The limitations are mostly related to the laborious methods involved (not to mention that after each synthetic step some purification is required) and the low yields.

## Discussion and concluding remarks

The saponin QS-21 is an outstanding immunostimulant agent for vaccine formulation. However, the current dependence on the Chilean wild forest as source of raw material for QS-21 (and other saponin extracts) has raised concerns about the ecological impact of bark harvesting, and the reliability as well as the sustainability of the future supply of this saponin for pharmaceutical industry. However, those concerns must be scrutinized carefully in light of three facts:

- The process employed by Desert King, the largest producer of quillaja extracts in the world (3), for production of tensoactive extracts is based largely on the use of all the wood of the tree, preserving the use of bark only for pharmaceutical grade extracts. This approach, implemented successfully since 1996, allowed a sustainable and permanently growing market for quillaja extracts without impacting on the sustainability of the business (78).
- The collection of quillaja biomass from the wild forest is a highly regulated activity in Chile, monitored by the Corporación Nacional Forestal (CONAF), ensuring the protection of quillaja forests and the sustainability of the bark collection (79). The current method of bark and biomass collection from wild forests in Chile is a part of whole silvicultural management based on the thinning and pruning of the quillaja trees. The management of quillaja in wild sources, revitalizes the trees and prevents the degradation of the forests; additionally enhances the productivity of biomass and bark, allowing the recovery of harvested trees within a period of 10 to 12 years. The management of quillaja forests have other important side benefits; the vigorous development of the shoots allows the grazing of animals after two/three years of the harvest. As another interesting example, quillaja is being used in the production of monofloral honeys with high export potential and has become an alternative of real interest for forestry and agricultural plantations (80).
- Quillaja trees have a wide distribution throughout the country - longitudinal distribution from 30° to 38° S (**Figure 2**). Although the risk of wildfires must not be neglected, its impact is limited to specific areas of the country.

According to our analysis, the potential of the quillaja bark as raw material must not be neglected; properly managed the current source would allow the purification of substantial amounts of QS-21. According to a conservative estimate mentioned in the introduction of this article, the potential availability of dry bark from native forest is ~ 14, 600 Tons; considering the demand of ~ 200 Ton/year of bark - maximum sustainable harvesting of bark it is estimated in 600 Ton/year (3), current available raw material suffices for several kilograms/year of QS-21.

However, to achieve higher reliability of the sources of raw material, and to reduce the costs involved in selection of raw material for saponin purification, novel technologies are currently in development: plantations of clonal quillaja trees, and emerging biotechnological and synthetic approaches (plant cell culture of *Q. saponaria* cells, bioreactor culture of genetically engineered *S. cerevisiae* and also the chemical synthesis from quillaic acid as starting material). The reviewed production methods highlight the industry’s push toward sustainability and scalability in QS-21 supply. The potential of plantation model based on clonal trees is huge; it reduces the impact and dependence on the native forest, but at the same time allows to sustain significant CO_2_ fixation. It is expected that plantation technology set in the future a benchmark in pharmaceutical-grade saponin production. However, the industrial use of biomass harvested from plantations (physically and chemically different from bark) must be validated at large scale. There are several challenges involved, mostly related to the initial to the harvesting and extraction steps. Regarding the latter, there is a significant space to achieve improvements. Currently the most widespread extraction method to produce quillaja extracts at large scale is the maceration, where the raw material is dried, milled and leached with hot water or ethanol to produce an extract containing saponins (4, 78). Drawbacks of maceration are the long extraction times and the large amount of solvent used (81); the above may have a negative impact in the processing of fresh biomass harvested from a plantation, where a higher metabolic activity is expected. However, today there is a range of so-called green technologies, such as microwave-assisted and ultrasound-assisted extraction, aimed to reduce the use of solvents, energy consumption and time while increasing the recovery of plant metabolites (82). In microwave assisted extraction, microwaves penetrate into wet raw material interacting with polar molecules such as water being a local heating mechanism (82). Solvent-free microwave-assisted extraction methods are available and could be applied to saponin extraction (83). Ultrasound creates cavitation bubbles in the solvent that alter the structure of the raw material during extraction, increasing solvent penetration and solute recovery in less time and lower temperatures, making the process suitable for thermolabile and more unstable saponins (84). This technology may be further improved by addition of enzymes; an efficient ultrasound-assisted enzymatic method for extracting the QS-21 from quillaja bark treated with tannase was proposed by Gao et al. (85). Regarding the purification process applied to the quillaja extract, it is expected that some adjustments to the traditional method currently applied to bark must be applied, to allow the processing of fresh material produced in plantations (an exception is the bark harvested from clonal forestry plantations, where the current process applied to the bark collected from wild forests is expected to perform with equal efficiency).

Regarding the final chromatographic step of isolation of QS-21, it is expected that the use of clonal material from plantations (lacking saponin analogs eluting close to QS-21) would enhance significantly the yields of recovery of QS-21 (56).

Regarding the emerging biotechnological and synthetic approaches, plant cell culture systems derived from *Q. saponaria* have demonstrated proof-of-concept QS-21 biosynthesis with reported structural and immunological comparability to bark-derived material. Current reported titers are in the milligram-per-liter range, which would require substantial bioreactor capacity for industrial-scale implementation. As mentioned earlier, the bioreactor capacity estimated to purify 50 Kg/year of QS-21 is 54, 200 m^3^/year. That figure is huge; the world’s largest plant cell culture cGMP facility (situated in Ahrensburg, Germany) is used to produce paclitaxel on a 75 m^3^ scale (86). Assuming an operational cycle of 82 h per batch of *Q. saponaria* cells, at least 7 bioreactors of 75 m^3^ would be required operating in a non-stop mode throughout a year to produce 50 Kg of QS-21. Considering the yield reported by Lv et al. (38), it is unlikely that cell culture could compete with the production of QS-21 from bark or biomass from plantations unless significant improvements in the productivity of QS-21 are achieved. Engineered *Saccharomyces cerevisiae* strains capable of *de novo* QS-21 biosynthesis have also been described, representing a significant advance in pathway engineering. At present, reported yields remain limited, and further optimization of fermentation efficiency and downstream purification would be required to enable large-scale production. As mentioned earlier, 528, 541 m^3^/year of recombinant yeast culture would be required to produce enough raw material to isolate 50 Kg/year of QS-21, according to the available data (51). Although such volume is huge, it is still feasible for yeast fermentation (73). However, the extremely low concentration of QS-21 in the culture (just 94.6 mg/m^3^ for QS-21_Api_; by comparison raw bark extract contain at least 1, 000, 000 mg/m^3^ (61, 62) anticipate high costs of downstream processing during the purification stage. In spite of the anticipated issues, the technology is quite promising, and future improvements in the final titer of saponin by adjustment of culture conditions might transform this process in a competitive alternative to produce QS-21. Total chemical synthesis provides access to defined QS-21 isomers but involves complex multistep routes with low overall yield, which may affect cost and scalability under current methodologies. Currently, there is no known commercial project to produce synthetic QS-21 (3).

Collectively, these emerging approaches remain under active development, and their future industrial feasibility will depend on improvements in productivity, cost efficiency, and regulatory validation. While emerging technologies may eventually diversify the QS-21 supply chain, clonal plantation technology presently stands as the most promising (currently on validation stage), balanced and sustainable solution, optimizing quality, environmental stewardship, and commercial feasibility, and setting a benchmark for pharmaceutical-grade QS-21 production.

## Supplementary material

The Supplementary Material for this article can be found onlineat: https://www.frontiersin.org/articles/10.3389/fimmu.2026.1813847/full#supplementary-material
