# Supplementary material for: The challenge of one billion adjuvanted vaccine doses: evaluating scalability, sustainability, and supply capacity of Quillaja saponin QS-21 for large-scale vaccine demand
Source: Front Immunol. 2026 Apr 17;17:1813847. doi: 10.3389/fimmu.2026.1813847 (PMC13133571; doi:10.3389/fimmu.2026.1813847)
Supplement: Supplementary file 1 [file DataSheet1.docx]

**Supplementary information - The challenge of one billion adjuvanted vaccine doses: Evaluating scalability, sustainability, and supply capacity of Quillaja saponin QS-21 for large-scale vaccine demand.**

Leandro Padilla^1*^, Roberto Bobadilla^1^, Marcelo Orellana^1^, Javier González^1^, Damian Hiley^2^, Rodrigo Otero^2^, Zoltan Beck^2^.

^1^ Desert King Chile, Valparaíso, Chile.

^2^ Desert King International, San Diego, CA, United States.

* Correspondence: lpadilla@desertking.cl; Tel: +56-32-2925020.

**CALCULATIONS OF THE QS-21 PRODUCTION FROM SEVERAL SOURCES.**

The purpose of this section is to show the calculations performed to compare different sources in a scenario of yearly demand of 50 Kg of QS-21. Based on published data (1), that amount would suffice to produce 1,000,000,000 doses of Shingrix vaccine (GSK) containing 50 µg QS-21 each (currently this vaccine has the highest reported content of QS-21, allowing a conservative estimation of the demand QS-21/dose).

**Purification from extracts of selected Quillaja bark collected in the wild forest (traditional process).**

Based on literature sources, the yield of the purification process of QS-21 is ~ 1,700 Kg bark per Kg of QS-21 (2). To be conservative in our estimates, we rounded that value to 2,000 Kg of selected dry bark per Kg QS-21. The bark employed for processing must be previously characterized to determine its suitability for QS-21 production. To that end, the saponin analysis is performed by reverse phase UHPLC chromatography (3) in order to select bark batches with high content of QS-21 and low amounts of impurities interfering in the chromatographic purification of that saponin - **Supplementary Figure 1** shows UHPLC traces of extracts of suitable and unsuitable bark for QS-21 production.

**Supplementary Figure 1:** Reverse phase UHPLC characterization of bark very suitable (A), still suitable (B) and unsuitable (C) for QS-21 production. Unsuitable bark has a significant content of the preceding peak Rha-QS-21, the major interfering impurity in the chromatographic purification of QS-21 - chromatograms taken from Padilla-Iglesias *et al*. (3).

The total requirement of selected bark to produce 50 Kg of QS-21 is

$$50\frac{Kg QS21}{year}\cdot2,000\frac{Kg selected bark}{Kg QS21}=100,000 \frac{Kg selected bark}{year}$$

Considering that ~ 50% of harvested bark is selectable for QS-21 purification, the total requirement of harvested bark is

$$\frac{100,000 \frac{Kg selected bark}{year}}{0.5\frac{Kg selected bark}{Kg harvested bark}}=200,000 \frac{Kg harvested bark}{year}$$

The surplus of purchased bark not used as raw material for QS-21 production, could be employed in the manufacturing of other adjuvants.

Based on published data of content of bark in total biomass (11% w/w according to Copaja *et al*. (4)), the total content of carbon in tree biomass (50% of C on dry tree biomass according to Matthews (5)), and the molar masses of CO_2_ and C (44 and 12 g/mol respectively), the total CO_2_ fixation required to produce the harvested material is

$$\frac{200,000 Kg harvested bark}{0.11\frac{Kg bark}{Kg biomass}}\cdot0.5\frac{Kg C}{Kg biomass}\cdot\frac{44 \frac{g {CO}_{2}}{mol}}{12 \frac{g}{mol}}=3,333,333 Kg {CO}_{2}$$

**Purification from extracts of bark harvested from clonal quillaja trees grown in plantations.**

This technology is based on the harvesting of bark from plantations of clone trees of suitable chemotype at a density of 1,000 plants/hectare, grown during 15 years. **Supplementary Figure 1A** shows the UHPLC trace of a bark extract of such chemotype having a negligible content of the preceding peak Rha-QS-21, the major interfering impurity in the chromatographic purification of QS-21 (3). The cloning of selected chemotypes of *Q. saponaria* is described by Padilla-Iglesias *et al*. (3); the method allows the production of thousands of clone plants suitable for cultivation in plantations. All the bark harvested in a clonal plantation of such chemotype is chemically homogeneous and suitable for QS-21 purification; therefore, raw material selection after harvesting is no longer needed.

Again, the yield of the purification process is expected to be ~ 1 Kg QS-21 per 2,000 Kg of harvested dry bark; therefore, the total requirement of bark to produce 50 Kg of QS-21 is calculated as follows:

$$50\frac{Kg QS21}{year}\cdot2,000\frac{Kg harvested bark}{Kg QS21}=100,000 \frac{Kg harvested bark}{year}$$

Under the weather conditions in the central area of Chile, quillaja trees is expected to be suitable for harvesting after 15 years of growth, rendering ~ 6,250 Kg of bark per hectare. To produce that amount of bark every year, the planted surface required is calculated as follows:

$$\frac{100,000 Kg harvested bark}{6,250\frac{Kg harvested bark}{hectare}}=16 hectares$$

Upon harvesting of trees, the stumps are able to regrow; it is expected that after 15 years one hectare will produce again ~ 6,250 Kg of bark per hectare. Fifteen land pads of 16 hectares each (total surface, 240 hectares), planted consecutively on 15 years can be harvested (one per year) in order to secure continuous supply of raw material over the years.

Similarly to the calculation performed for traditional technology case, the total CO_2_ fixation required to produce the harvested material is calculated as follows:

$$\frac{100,000 Kg harvested bark}{0.11\frac{Kg bark}{Kg biomass}}\cdot0.5\frac{Kg C}{Kg biomass}\cdot\frac{44 \frac{g {CO}_{2}}{mol}}{12 \frac{g}{mol}}=1,666,667 Kg {CO}_{2}$$

**Purification from extracts of aerial biomass harvested from clonal quillaja trees grown in ultra high density plantations.**

This technology is based on the harvesting of aerial biomass (without leaves) from plantations of clone trees of suitable chemotype at a density of 50,000 plants/hectare, grown only 3 years. **Supplementary Figure 2** shows the UHPLC trace of a biomass extract of such chemotype having a negligible content of the interfering peak Rha-QS-21 (3).

**Supplementary Figure 2:** Reverse phase UHPLC characterization of ultrahigh density biomass of a chemotype suitable for QS-21 production. This chemotype has negligible content of the preceding peak Rha-QS-21, the major interfering impurity in the chromatographic purification of QS-21 - chromatogram taken from Padilla-Iglesias *et al*. (3).

Similarly to forestry plantation, all the biomass harvested in an ultra-high density clonal plantation of such chemotype is chemically homogeneous and suitable for QS-21 purification; therefore, raw material selection after harvesting is no longer needed.

Since the overall content of QS-21 in the biomass is lower than in the bark, we estimate a yield of the purification process of ~ 1 Kg QS-21 per 7,480 Kg of dry harvested biomass; therefore, the total requirement of biomass to produce 50 Kg of QS-21 is calculated as follows:

$$50\frac{Kg QS21}{year}\cdot7,480\frac{Kg harvested biomass}{Kg QS21}=374,000 \frac{Kg harvested biomass}{year}$$

After 3 years of growth, the yield of dry biomass is ~ 17,000 Kg per hectare. To produce 374,000 Kg of dry biomass, the planted surface required is calculated as follows:

$$\frac{374,000 Kg harvested biomass}{17,000\frac{Kg harvested biomass}{hectare}}=22 hectares$$

Upon harvesting of plants, the stumps are able to regrow; it is expected that after 3 years one hectare will produce again ~17,000 Kg dry biomass per hectare. Three land pads of 22 hectares (total surface 66 hectares) planted consecutively on 3 years can be harvested (one per year) in order to secure continuous supply of raw material over the years.

Considering that the content of leafless biomass on total biomass is 75.6% w/w (3), and similarly to the calculations performed for processing of bark, the total CO_2_ fixation required to produce the harvested biomass is calculated as follows:

$$\frac{374,000 Kg harvested leafless biomass}{0.756\frac{Kg leafless biomass}{Kg biomass}}\cdot0.5\frac{Kg C}{Kg biomass}\cdot\frac{44 \frac{g {CO}_{2}}{mol}}{12 \frac{g}{mol}}=906,966 Kg {CO}_{2}$$

***De novo* synthesis of QS-21 in cultures of *Q. saponaria* cells.**

According to Lv *et al*. (6) 26 L of culture of *Q. saponaria* cells rendered 250 g of dry biomass containing an average of 2,700 mg QS-21/Kg. Upon purification of the biomass material, 24 mg of pure QS-21 were recovered. Assuming equivalent yields at larger scale, the total volume of culture required to purify 50 Kg of QS-21 is calculated as follows:

$$50\frac{Kg QS21}{year}\cdot\frac{26 L of culture}{24\cdot{10}^{-6} Kg QS21}=54,166,667 \frac{L of culture}{year}$$

Lv *et al*. (6) did not provide data to estimate the CO_2_ fixation associated to the production of biomass.

***De novo* synthesis of QS-21 in cultures of *S. cerevisiae* cells engineered with genes involved in its biosynthesis.**

According to Liu *et al*. (7), a culture of engineered cells of *S. cerevisiae* YL-46 produces a liquid broth containing 94.6 μg/L of the isomer QS-21-Xyl; QS-21 can be isolated by chromatographic means from that broth. Assuming a total recovery of QS-21 from the broth (Liu *et al*. (7) did not provide the purification yield), the total volume of culture required to purify 50 Kg of QS-21 is calculated as follows:

$$50\frac{Kg QS21}{year}\cdot\frac{1 L of culture}{94.6\cdot{10}^{-9} Kg QS21}=528,541,226 \frac{L of culture}{year}$$

On the other hand, a culture of engineered cells of *S. cerevisiae* YL-47 produces a liquid broth containing 31.1 μg/L of the isomer QS-21-Api. Similarly to the calculation for QS-21-Xyl isomer, the total volume of culture required to purify 50 Kg of QS-21 is calculated as follows:

$$50\frac{Kg QS21}{year}\cdot\frac{1 L of culture}{31.1\cdot{10}^{-9} Kg QS21}=1,607,717,042 \frac{L of culture}{year}$$

Since the growth of *S. cerevisiae* is heterotrophic, no CO_2_ fixation is associated to the production of biomass and QS-21.

**Chemical synthesis of QS-21: Glycosylation/acylation of quillaic acid from natural sources.**

This technology is based on the glycosylation/acylation of quillaic acid produced by acid hydrolysis of quillaja saponins – according to the descriptions of Cartagena (8) (production of quillaic acid from Type 2 Quillaja extract), Wang *et al*. (9) (synthesis of QS-21_Api_) and Deng *et al*. (10) (synthesis of QS-21_Xyl_). **Supplementary Tables 1** and **2** summarize the yields of the chemical synthesis of both QS-21 isomers.

**Supplementary Table 1:** Yields of the chemical synthesis of QS-21_Api_ from Quillaja extract Type 2.

| **Step** | **Reaction** | **Yield**  **[% w/w]** | **Source** |
| --- | --- | --- | --- |
| 1 | Acid hydrolysis of Type 2 Quillaja extract to produce quillaic acid | 0.95% | Figure 3 in Cartagena (8) |
| 2 | Allylation of quillaic acid with allyl bromide (carboxyl allylation) | 70.0% | Scheme 3a in Wang *et al*. (9) |
| 3 | Glycosylation of 3-O position of allyl-quillaic acid with protected trisaccharide to render allyl-prosapogenin | 59.0% | Scheme 3a in Wang *et al*. (9) |
| 4 | Deallylation of allyl prosapogenin | 74.5% | Scheme 3a in Wang *et al*. (9): Two consecutive steps with yields 92% and 81% |
| 5 | Addition to 28-O of prosapogenin position of protected tetrasaccharide+Fa-Ara chain | 70.0% | Scheme 3a in Wang *et al*. (9) |
| 6 | Production of QS-21_Api_ from protected precursor | 75.0% | Scheme 3a in Wang *et al*. (9) |
|  | **Total yield of QS-21_Api_** | **0.154%** | **Calculated as the product of 1-6 steps** |

**Supplementary Table 2:** Yields of the chemical synthesis of QS-21_Xyl_ from Quillaja extract Type 2.

| **Step** | **Reaction** | **Yield**  **[% w/w]** | **Source** |
| --- | --- | --- | --- |
| 1 | Acid hydrolysis of Type 2 Quillaja extract to produce quillaic acid | 0.95% | Figure 3 in Cartagena (8) |
| 2 | Allylation of quillaic acid with allyl bromide (carboxyl allylation) | 70.0% | Scheme 3a in Wang *et al*. (9) |
| 3 | Glycosylation of 3-O position of allyl-quillaic acid with protected trisaccharide to render allyl-prosapogenin | 59.0% | Scheme 3a in Wang *et al*. (9) |
| 4 | Deallylation of allyl prosapogenin | 74.5% | Scheme 3a in Wang *et al*. (9): Two consecutive steps with yields 92% and 81% |
| 5 | Addition to 28-O of prosapogenin position of protected tetrasaccharide+Fa-Ara chain | 80.0% | Scheme 4 in Deng *et al*. (10) |
| 6 | Production of QS-21_Xyl_ from protected precursor | 64.0% | Scheme 4 in Deng *et al*. (10) |
|  | **Total yield of QS-21_Xyl_ from quillaic acid** | **0.150%** | **Calculated as the product of 1-6 steps** |

The yields of both isomers are quite similar; taking the slightly lower yield of QS-21_Xyl_, the total amount of quillaja extract Type 2 required to produce 50 Kg of QS-21 is calculated as follows:

$$50\frac{Kg QS21}{year}\cdot\frac{100}{0.15}\frac{Kg Type 2 extract}{Kg QS21}=33,333 \frac{Kg Type 2 extract}{year}$$

The production of 33 Ton/year of Type 2 extract containing 65-90% saponins (11) would require 3,282 Ton/year of fresh biomass (wood + bark) from adult trees (12). Assuming 40% w/w of moisture content in the biomass, the total CO_2_ fixation required to produce the harvested material is calculated as follows:

$$3,282,000 Kg fresh biomass\cdot0.6\frac{Kg biomass}{Kg fresh biomass}\cdot0.5\frac{Kg C}{Kg biomass}\cdot\frac{44 \frac{g {CO}_{2}}{mol}}{12 \frac{g}{mol}}=3,610,200 Kg {CO}_{2}$$

**REFERENCES OF THE SUPPLEMENTARY INFORMATION.**

1. Treatment Action Group. From forest to factory: Analyzing the supply chains for two modern adjuvants of global health importance – QS-21 and MPL. New York: Treatment Action Group (2025). 79 p.
2. San Martín R, Liu Y, Yang Y, Fox CB, Iyer LR, et al. Structure and immunological activity of QS-21 variant from *Quillaja saponaria* aerial biomass. Front Immunol (2026) 17:1771912. doi: 10.3389/fimmu.2026.1771912.
3. Padilla-Iglesias L, González-Castro J, Otero-Peredo R, inventors; Desert King Chile, assignee. Production of biomass in ultra high density plantations. United States patent US 11254699 (2022).
4. Copaja SV, Blackburn C, Carmona R. Variation of saponin contents in *Quillaja saponica* Molina. Wood Sci Technol (2003) 37:103-108. doi: 10.1007/s00226-002-0150-8.
5. Matthews GAR. The carbon content of trees. Edinburgh, UK: UK Forestry Commission (1993). 28 p.
6. Lv X, Martin J, Hoover H, Joshi B, Wilkens M, Ullisch DA, et al. Chemical and biological characterization of vaccine adjuvant QS-21 produced via plant cell culture. iScience (2024) 27:109006. doi: 10.1016/j.isci.2024.109006.
7. Liu Y, Zhao X, Gan F, Chen X, Deng K, Crowe SA, et al. Complete biosynthesis of QS-21 in engineered yeast. Nature (2024) 629:937-944. doi: 10.1038/s41586-024-07345-9.
8. Cartagena-Ramírez C. (2010). Sapogeninas de un extracto de corteza de *Quillaja saponaria* Mol: Aislamiento, identificación y evaluación de potencial actividad hipoglicemiante *in-vitro*. [undergraduate thesis]. [Chile]: Universidad de Chile.
9. Wang P, Kim YJ, Navarro-Villalobos M, Rohde BD, Gin DY. Synthesis of the potent immunostimulatory adjuvant QS-21A. J Am Chem Soc (2005) 127:3256-3257. doi: 10.1021/ja0422007.
10. Deng K, Adams MM, Damani P, Livingston PO, Ragupathi G, Gin DY. Synthesis of QS-21-xylose: Establishment of the immunopotentiating activity of synthetic QS-21 adjuvant with a melanoma vaccine. Angew Chem Int Ed Engl (2008) 47:6395-6398. doi: 10.1002/anie.200801885.
11. Resnik S, Kuznesof PM, Valente Soares LM. Quillaia extracts: Type 1 and type 2. Paper presented at the 65th Meeting of the Joint FAO/WHO Expert Committee on Food Additives (JECFA), Geneva, Switzerland (2005).
12. Padilla-Iglesias L, Valencia-Michaud A, inventors; Desert King Chile, assignee. Method for obtaining saponins from plants United States patent US10660924 (2020).
